# A Call to Action: the Need for Standardization in Developing Open-Source Mass Spectrometry-Based Methods for Microbial Subspecies Discrimination

**DOI:** 10.1128/mSystems.00813-19

**Published:** 2020-02-18

**Authors:** Chase M. Clark, Brian T. Murphy, Laura M. Sanchez

**Affiliations:** a Department of Pharmaceutical Sciences, University of Illinois at Chicago, Chicago, Illinois, USA

**Keywords:** MALDI-TOF MS, bioinformatics, dereplication, microbial ecology

## EDITORIAL

In the last decade, there has been a renewed push by academic researchers to create rapid and accurate techniques to differentiate, identify, and prioritize culturable microbial isolates. One such technique that continues to gain momentum among microbiologists is matrix-assisted laser desorption–ionization time of flight mass spectrometry (MALDI-TOF MS). It is an established, inexpensive technique commonly used to rapidly identify microbial taxa and differentiate culturable microbes. This technology has become commonplace in clinical and veterinary laboratories where rigorously validated methods are used in conjunction with commercially available reference databases to identify pathogenic microorganisms. However, the broader community, especially laboratories working with environmental microbes, typically cannot access the expensive software and databases. It is our opinion that this community, which relies on free and open-source software, currently lacks a coherent set of accepted experimental practices, including employment of internal standard strains, statistically driven determination of biological and technical replicates, and deposition of MS data into open-access repositories. Establishing guidelines would enable researchers to better compare microbial typing methods and advance our ability to group and delineate environmental isolates in an effective manner, particularly at the subspecies level.

Toward this end, we recommend that future studies should, at a minimum, employ the following guidelines. (i) When creating/validating methods, the reported accuracy/precision should be obtained using spectra from new biological replicates or closely related strains (“test data”) that were not used to determine the parameters of the method (“training and validation data”). Though often a challenge, test spectra should be acquired using a different instrument/laboratory than was used to acquire training spectra. (ii) In addition to reporting culture duration, MALDI matrix type, and other similar variables, published studies should report the experimental design of collected data, including biological and technical replication, randomization, and blocking (designed to account for sources of variation and confounding, such as sample location on the MALDI target plate, day of data collection, chemical/medium ingredient batches, etc.) (1). (iii) Published studies should make data available for public use in both raw and standard open format (e.g. mzML) (2) in a repository such as the Mass Spectrometry Interactive Virtual Environment data repository (MassIVE; https://massive.ucsd.edu) (3).

We strongly recommend that guidelines ii and iii be considered for all microbially based MALDI-TOF MS publications, not just method development studies. Also, in addition to the aforementioned guidelines, the microbiology community would benefit from standardized and validated benchmark data sets, analogous to the metagenomics CAMI data sets and standards (4). For benchmarking, we suggest following a pattern of logic similar to that of Vervier et al. (5) and hope to see further participation of corporations in creating open standards and data (6).

Lastly, we urge caution when benchmarking methods that were trained/validated on different data sets. Assertions such as “exceeding the taxonomic resolution of other methods” (7) must be made with caution, as there are many appropriate approaches to dereplicate/differentiate microorganisms using MALDI-TOF MS. While many methods continue to focus solely on protein *m/z* regions, we previously developed a freely available, open-source MALDI-TOF MS-based pipeline (IDBac) to group bacterial isolates by protein MS spectra (2 to 20 kDa) in addition to specialized metabolite MS spectra (<2 kDa), allowing us to achieve rapid and accurate subspecies dereplication of environmental microbial isolates (8–10). Therefore, when attempting to achieve subspecies resolution of isolates, we opine that it is advantageous to characterize microorganisms using as many orthogonal methods (metabolomics, genomics, proteomics) as possible. It is an exciting time for MALDI-TOF MS analytical and technical innovation, and in our opinion, we have only scratched the surface of its usefulness for both proteomics and metabolomics. Looking forward, establishing global reference data sets in addition to community standards for data analysis, data sharing, and method comparison will result in more accurate assessments of our ability to distinguish microbial strains at the subspecies level.
